# Rab8 Binding to Immune Cell-Specific Adaptor LAX Facilitates Formation of *trans*-Golgi Network-Proximal CTLA-4 Vesicles for Surface Expression

**DOI:** 10.1128/MCB.01331-13

**Published:** 2014-04

**Authors:** Matthew C. Banton, Kerry L. Inder, Elke Valk, Christopher E. Rudd, Helga Schneider

**Affiliations:** a Cell Signalling Section, Division of Immunology, Department of Pathology, Cambridge University, Cambridge, United Kingdom; b Cambridge Institute for Medical Research, Cambridge, United Kingdom

## Abstract

Despite playing a central role in tolerance, little is known regarding the mechanism by which intracellular CTLA-4 is shuttled from the *trans*-Golgi network to the surfaces of T cells. In this context, Ras-related GTPase Rab8 plays an important role in the intracellular transport, while we have previously shown that CTLA-4 binds to the immune cell adaptor TRIM in T cells. In this study, we demonstrate that CTLA-4 forms a multimeric complex comprised of TRIM and related LAX that in turn binds to GTP bound Rab8 for post-Golgi transport to the cell surface. LAX bound via its N terminus to active GTP-Rab8, as well as the cytoplasmic tail of CTLA-4. TRIM required LAX for binding to Rab8 in a complex. Wild-type LAX or its N terminus (residues 1 to 77) increased CTLA-4 surface expression, whereas small interfering RNAs of Rab8 or LAX or disruption of LAX/Rab8 binding reduced numbers of CTLA-4-containing vesicles and its coreceptor surface expression. LAX also promoted the polarization of CTLA-4 and the reorientation of the microtubule-organizing center to the site of T-cell receptor engagement. Our results identify a novel CTLA-4/TRIM/LAX/Rab8 effector complex in the transport of CTLA-4 to the surfaces of T cells.

## INTRODUCTION

The T-cell coreceptor cytotoxic T-cell antigen 4 (CTLA-4) functions as a critical inhibitory regulator of T-cell responses. Its importance is demonstrated by the autoimmune phenotype of CTLA-4-deficient mice where polyclonal T-cell proliferation leads to massive tissue infiltration and early lethality due to organ destruction (1, 2). In addition, small nucleotide polymorphisms in the human CTLA-4 gene region have been implicated in numerous autoimmune disorders, including type 1 diabetes (3). Several mechanisms have been reported to account for CTLA-4 inhibition, including cell intrinsic and extrinsic pathways. Intrinsic pathways include inhibition of ZAP-70 microcluster formation (4), disruption of CD28 localization at the immunological synapse (5), and alteration of T-cell receptor (TCR) signaling by associated phosphatases SHP-2 and protein phosphatase 2A (PP2A) (6, 7), as well as interference with mechanisms such as ectodomain competition for CD28 binding to CD80 and CD86 (8) or their downregulation or removal by CTLA-4 (9) and the release of indoleamine 2,3-dioxygenase (10) and transforming growth factor β (11). Further, we have shown that CTLA-4 ligation activates the small GTPase Rap-1, leading to an increase in LFA-1-induced cell adhesion and motility (12, 13). Increased cell motility potently reduces the TCR-mediated stop signal and contact times with antigen-presenting cells, resulting in attenuated T-cell activation (12–15). The induction of motility has been confirmed by several laboratories (16–18).

Despite its importance for properly controlled immune responses, surface expression of CTLA-4 is tightly regulated with the majority of the coreceptor being localized in intracellular compartments such as the *trans*-Golgi network (TGN), endosomes, and lysosomes (19–23). The mechanism(s) regulating CTLA-4 surface expression has been unclear. Activated T cells in the lymphoproliferative disease Chediak-Higashi syndrome (CHS) lacking CTLA-4 expression have been proposed to play a role in CHS (24). Trafficking of CTLA-4 from the TGN to the plasma membrane is mediated by binding to the immune cell-specific type I transmembrane adapter TRIM (T-cell receptor-interacting molecule) (22, 25). Downregulation of TRIM significantly reduces CTLA-4 surface expression while retaining the coreceptor in the TGN (22, 25). In contrast, increased TRIM expression releases CTLA-4 to the cell surface, resulting in enhanced suppression of T-cell activation. On the cell surface, phosphorylated CTLA-4 binds to the lipid kinase phosphatidylinositol 3-kinase (PI3K) (26), while the nonphosphorylated CTLA-4 associates with the clathrin adapter complex AP-2 (20, 27–30). AP-2 mediates the internalization of CTLA-4 to intracellular compartments, whereas AP-1 regulates trafficking of CTLA-4 by shuttling the receptor from the TGN to lysosomes (20, 31).

Immune cell-specific transmembrane adaptor proteins such as LAT (linker for activation of T cells), SIT (SHP2 interacting transmembrane adapter protein), and LAX (linker for activation of X cells) are characterized by having a truncated non-ligand-binding extracellular domain, a transmembrane region, and an extended cytoplasmic tail (32). The TCR zeta chain is needed for the transport of the antigen receptor (TCR) to the cell surface (33). A similar role has been defined with TRIM in its promotion of the surface expression of CTLA-4 (22). Together, these observations have begun to implicate transmembrane type I adaptor proteins in the transport of cargo to the cell surface. TRIM-deficient mice also display no apparent defect in immune function (34), suggesting that other transmembrane adaptors may compensate for the absence of TRIM (34). At the same time, the non-raft-associated transmembrane adaptor proteins SIT and LAX act as negative regulators of T-cell activation (35, 36). There has been no evidence to indicate that either of these adaptors is involved in intracellular trafficking.

Besides adaptor proteins, small GTPases of the Rho family control the formation of lamellipodia, filopodia, and focal adhesions during cell morphogenesis (37). Of these, Rab proteins are members of the Ras superfamily that contain a conserved GTP/GDP-binding site. Furthermore, Rab proteins are described as regulators of protein transport of the secretory and endocytic pathways (38, 39). Rab5 is reported to control membrane trafficking from the plasma membrane to early endosomes (40). Many effectors, such as EEA1 and rabenosyn 5, are involved in this pathway (41–43). In contrast, Rab8 has been linked to the organization of the endocytic compartment (44), where it localizes to the endosomal recycling compartment (45, 46) and colocalizes with EHD1 (EH domain-containing protein 1) (47) and partially with Rab11 (44). Rab8 alters the reorganization of actin and microtubules, as well as directing membrane transport to cell surfaces (48, 49). Depletion of Rab8 promotes the formation of actin stress fibers, whereas its activation can lead to cell protrusions (44, 50). Further, Rab8 interacts via optineurin with myosin Vb (51) and has also been reported to bind mitogen-activated protein kinase kinase kinase kinase 2 (MAP4K2) (52), the coiled-coil protein FIP-2 (53), and rab8ip/GC kinase, a participant in tumor necrosis factor alpha-mediated signaling pathway (52). In addition, the α_2_B- and β_2_-adrenergic receptors have been described to bind Rab8 for transport to the plasma membrane (54). Despite its high expression in T cells, no immune cell-specific binding effectors of Rab8 have been identified.

While we previously showed that CTLA-4 binds to the immune adaptor TRIM in T cells, the exact nature of the transport complex has been unclear. In the present study, we demonstrate that CTLA-4 forms a multimeric complex comprised of TRIM and related LAX that binds to GTP bound Rab8 for post-Golgi transport to the cell surface. Wild-type LAX (LAX WT) or its N terminus (residues 1 to 77) increased CTLA-4 surface expression, whereas small interfering RNAs (siRNAs) of Rab8 and LAX reduced the numbers of CTLA-4-containing vesicles and its coreceptor surface expression. Our results identify a novel CTLA-4/TRIM/LAX/Rab8 effector complex in the transport of CTLA-4 to the surfaces of T cells.

## MATERIALS AND METHODS

### Reagents and plasmids.

Anti-myc and alpha-tubulin antibodies were purchased from Millipore, antihemagglutinin (anti-HA) from Covance, anti-SIT from BioLegend, anti-LAT from Upstate Biotechnology, anti-TRIM from Abcam, anti-syntaxin-6 and Rab8 from BD Bioscience, anti-CD3 (145-2C11) from BioXCell (West Lebanon, NH), anti-human CTLA-4 conjugated to phycoerythrin (PE) and mouse IgG2a conjugated to PE from BD Pharmingen, anti-V5 and Alexa-conjugated secondary antibodies from Invitrogen, and horseradish peroxidase (HRP)-conjugated secondary antibodies from Jackson Immuno-Research. Anti-mouse CTLA-4 was purchased from Santa Cruz. Anti-human CD28 (9.3) was from Bristol Meyers Squibb, and anti-human CTLA-4 (BNI3) and polyclonal anti-LAX antibodies were kindly provided by B. Bröker (Greifswald, Germany) and W. Zhang (Durham, NC), respectively.

CTLA-4, CD28, and TRIM plasmids have been described elsewhere (20, 22). pcDNA3 CTLA-4 tailless was a gift from A. Hueber (Erlangen, Germany). pCEFL myc-LAX and pEF6 myc-His-LAX were kindly provided by W. Zhang (Durham, NC) and V. Shapiro (Rochester, NY), respectively. pEGFP myc-Rab8 wild type, pEGFP-Rab8Q67L, pEGFP-Rab8T22N, and pEGFP-Rab27 were kindly provided by J. Peränen (Helsinki, Finland). pcDNA3 myc-LAT was a generous gift from L. Samelson (Bethesda, MD). A plasmid containing full-length SIT cDNA (Open Biosystems) was amplified by PCR using the primers 5′-CACCATGAACCAGGCTGACCC-3′ and 3′-TCACAGATCCTCTTCTGAGATGAGTTTTTGTTCGCTGGCTGCAGGCTG-5′. A C-terminal myc tag was cloned into the pcDNA3.1 directional TOPO expression vector (Invitrogen). LAX1-77 and LAX1-75 mutants were generated by PCR from the LAX plasmid using the same forward primer but different reverse primers: LAX forward, 5′-GCGGATCCCCTGATACAATGGATGG-3′; LAX1-77 reverse, 5′-GCTCTAGACATGACGGTAACTCGGAGGTAAG-3′; and LAX1-75 reverse, 5′-GCTCTAGAGGTAACTCGGAGGTAAGGAACTTG-3′. PCR products were digested using BamHI and XbaI and cloned into the pEF6 myc-His plasmid (Invitrogen). LAX- and Rab8-specific siRNAs (catalog numbers M-064303-00-0005 and M-040860-00-0005, respectively) consisting of four siRNA duplexes for each gene, and control nontargeting siRNAs (catalog number D-001206-13-05) were purchased from Dharmacon (Lafayette, CO).

### Cells and transfection.

DC27.10 cells stably transfected with CTLA-4 (DC27.10–CTLA-4) were cultured and transfected as described previously (20, 22). 293T cells, grown in Dulbecco modified Eagle medium with 10% fetal bovine serum, were transfected using Lipofectamine 2000 (Invitrogen). Murine T cells were transfected with LAX siRNA or Rab8 siRNA and controls (600 nM) using an Amaxa Nucleofector kit (Lonza, Germany).

### IL-2 assay.

DC27.10–CTLA-4 cells were transfected with control (mock), LAT, LAX, or TRIM and stimulated with plate-bound anti-CD3 (1 μg/ml) or anti-CD3/CTLA-4 (1 and 10 μg/ml, respectively). After 24 h, supernatants were taken, and the interleukin-2 (IL-2) concentration determined by enzyme-linked immunosorbent assay (ELISA; BD Biosciences) according to the manufacturer's protocol.

Transfected primary murine T cells were stimulated with plate-bound anti-CD3 (2.5 μg/ml) or anti-CD3/CTLA-4 (2.5 and 10 μg/ml, respectively). After 48 h, intracellular staining for IL-2 was performed as described previously (12).

### Immunoprecipitation and immunoblotting.

Immunoprecipitation and blotting were performed as previously described (20, 22). Briefly, cells were lysed in ice-cold lysis buffer (1% Triton X-100, 20 mM Tris, 150 mM NaCl [pH 8.0]) containing protease and phosphatase inhibitors. Postnuclear lysates were incubated for 1 h with the indicated antibody. Protein A- or G-Sepharose beads (GE Healthcare) were added, followed by incubation for 1 h at 4°C. The eluted proteins were separated by SDS-PAGE and transferred onto polyvinylidene difluoride membrane for immunoblotting. Membranes were blocked with 5% milk in phosphate-buffered saline (PBS)–1% Tween and incubated with the indicated antibody for 1 h. Bound antibody was revealed with the appropriate secondary antibody and protein was visualized by enhanced chemiluminescence using ECL (GE Healthcare).

### Immunofluorescence.

Cells were harvested, washed in PBS and fixed in Cytofix (BD Bioscience) for 30 min at 4°C. Cells were then permeabilized in PBS containing 0.5% saponin and 1% bovine serum albumin (BSA) for 90 min with primary antibody for 1 h. Cells were then washed in PBS containing 0.1% saponin and 1% BSA and incubated with fluorescently labeled secondary antibody for 1 h. Incubation and washing for subsequent primary and secondary antibodies was repeated for double- and triple-stained cells. After the final wash, cells were resuspended in Vectashield mounting medium containing DAPI (4′,6′-diamidino-2-phenylindole; Vector Laboratories), mounted onto microscope slides, and sealed with nail varnish. Image acquisition was performed with a Zeiss LSM510 confocal microscope using an ×63 PlanApochromat/1.4numerical-aperture oil objective lens. Lasers of 405 (DAPI)-, 488 (Alexa Fluor 488)-, 543 (Alexa Fluor 568)-, and 633 (Alexa Fluor 647)-nm wavelengths were used for fluorescence excitation. Images were analyzed using Fiji.

### MTOC reorientation assay.

Transfected DC27.10–CTLA-4 cells were seeded onto poly-d-lysine (Sigma-Aldrich)-coated coverslips in six-well plates. After binding, anti-CD3 (145-2C11)- or isotype-coated Dynabeads (4.5 μm in diameter [Invitrogen], prepared according to the manufacturer's instructions) were mixed with the cells at a 1:1 ratio, followed by incubation at 37°C for 15, 30, and 60 min. Cells were then washed and fixed in 4% paraformaldehyde for 10 min, washed thrice in PBS, permeabilized with 0.1% Triton X-100 for 10 min, washed twice in PBS, and blocked with 3% BSA in PBS for 1 h. Cells were then incubated with the respective antibodies. Washing and antibody incubation was repeated for subsequent antibodies in double- and triple-stained cells. After the final wash, cells on coverslips were mounted onto microscope slides with Vectashield mounting medium containing DAPI (Vector Laboratories). Microtubule-organizing center (MTOC) reorientation was determined by observing the samples with a Zeiss Axiophot fluorescence microscope and counting the number of cells whose MTOC position (as determined by tubulin staining) was juxtaposed to a bead in contact with the cell. Polarized MTOC was defined by being within 3 μm from the cell-bead contact area.

## RESULTS

### Rab8 colocalizes with and binds to the immune specific adaptor LAX.

**FIG 1:**
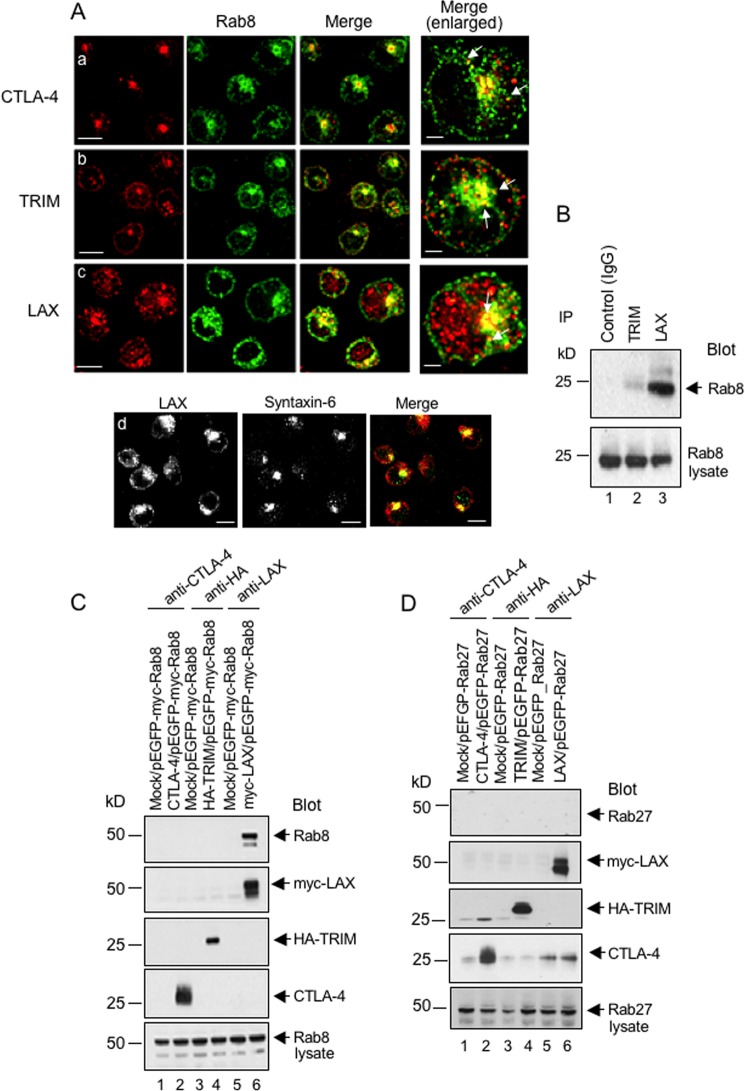
Rab8 colocalizes with CTLA-4, LAX, and TRIM. (A) Preactivated human T cells were stained with anti-CTLA-4 plus Alexa Fluor 568, followed by anti-Rab8 plus Alexa Fluor 488 (a), anti-TRIM plus Alexa Fluor 568 (b), or with anti-LAX plus Alexa Fluor 568 (c) and analyzed for colocalization (yellow, merged images) by confocal microscopy. Bar, 10 μm. The enlarged image shows a representative cell for colocalization with Rab8 and CTLA-4 (top), TRIM (middle), and LAX (bottom). Bar, 2.5 μm. (d) DC27.10–CTLA-4 cells were stained with anti-LAX, followed by anti-rabbit antibody–Alexa Fluor 568. In addition, cells were stained with anti-syntaxin-6, a marker for the Golgi apparatus, and assessed by confocal microscopy (TGN, green; LAX, red). Bar, 10 μm. (B) LAX binds to Rab8. Jurkat cells were immunoprecipitated with control antibody (IgG, lane 1), anti-TRIM (lane 2), or anti-LAX (lane 3) and blotted with anti-Rab8 MAb. Anti-Rab8 blotting of cell lysates served as a loading control (bottom panel). (C) LAX, but not TRIM, specifically binds to Rab8 and not to Rab27. 293T cells cotransfected with as indicated were immunoprecipitated with anti-CTLA-4 (lanes 1 and 2), anti-HA (lanes 3 and 4), or anti-LAX (lanes 5 and 6). Immunoblots were probed with anti-Rab8 (top panel), anti-myc (second panel), anti-HA (middle panel), or anti-CTLA-4 (fourth panel). Anti-Rab8 blotting of cell lysates served as a loading control (bottom panel). (D) 293T cells cotransfected as indicated were immunoprecipitated with anti-CTLA-4 (lanes 1 and 2), anti-HA (lanes 3 and 4), or anti-LAX (lanes 5 and 6). Immunoblots were probed with anti-Rab27 (top panel), anti-myc (second panel), anti-HA (middle panel), or anti-CTLA-4 (fourth panel). Anti-Rab27 blotting of cell lysates served as a loading control (bottom panel).

We previously found that immune cell adaptor TRIM associates with CTLA-4 and is needed for its transport to the surfaces of T cells (22). The small GTPase Rab8 has been reported to be involved in the trafficking of newly synthesized proteins from the TGN to the plasma membrane (55). However, a possible role of Rab8 and associated immune cell-specific adaptors in this process has been unclear. To assess the role of Rab8 on CTLA-4 expression, we first investigated colocalization of CTLA-4 with Rab8 and immune cell adaptors TRIM or LAX (Fig. 1A). For this, preactivated primary T cells were stained for endogenous protein using anti-CTLA-4, anti-LAX or anti-TRIM in combination with an Alexa Fluor 568-coupled secondary antibody, followed by anti-Rab8 plus Alexa Fluor 488. As we previously reported, CTLA-4 and TRIM stained the TGN and surrounding vesicles (Fig. 1Aa and b) (22). Intriguingly, anti-LAX stained the same region (Fig. 1Ac) that colocalized with the syntaxin-6 staining of the TGN (Fig. 1Ad). Anti-Rab8 also stained the TGN and surrounding vesicles, whereas a subset of Rab8 vesicles stained positive for CTLA-4 (upper panel, see arrows and merged single slice figure). A similar pattern of colocalization was observed between Rab8 and TRIM (middle panel) and LAX (lower panel). These data suggested a potential interaction between CTLA-4, TRIM, and/or LAX and Rab8 in the TGN and proximal vesicles.

Given this colocalization, we sought to determine whether Rab8 could bind CTLA-4, TRIM, and/or LAX (Fig. 1B). Thus far, no immune cell-specific adaptors have been reported to associate with Rab8. Jurkat T cells were therefore lysed and subjected to precipitation with anti-TRIM or anti-LAX, followed by immunoblotting with anti-Rab8. Although anti-LAX coprecipitated Rab8 (Fig. 1B, lane 3), anti-TRIM precipitated only little Rab8 (Fig. 1B, lanes 2). As a control, cell lysates blotted with anti-Rab8 showed equal sample loading (Fig. 1B, lower panel, lanes 1 to 3).

To confirm this observation by cotransfection in nonlymphoid cells, mock, CTLA-4, HA-TRIM, or myc-LAX were coexpressed with pEGFP-myc-Rab8 in 293T cells, followed by precipitation with anti-CTLA-4, anti-HA, or anti-LAX and blotting with anti-Rab8 (Fig. 1C, lanes 1 to 6). Although CTLA-4 and TRIM failed to bind to Rab8 (Fig. 1C, lanes 2 and 4), anti-LAX readily precipitated Rab8 from cells cotransfected with myc-LAX and pEFGP-myc-Rab8 (Fig. 1C, lane 6). As a further control, neither anti-CTLA-4, TRIM, nor LAX coprecipitated Rab27 from cells cotransfected with pEGFP-Rab27 (Fig. 1D). These data indicated that LAX, but neither CTLA-4 nor TRIM, bound to Rab8.

**FIG 2:**
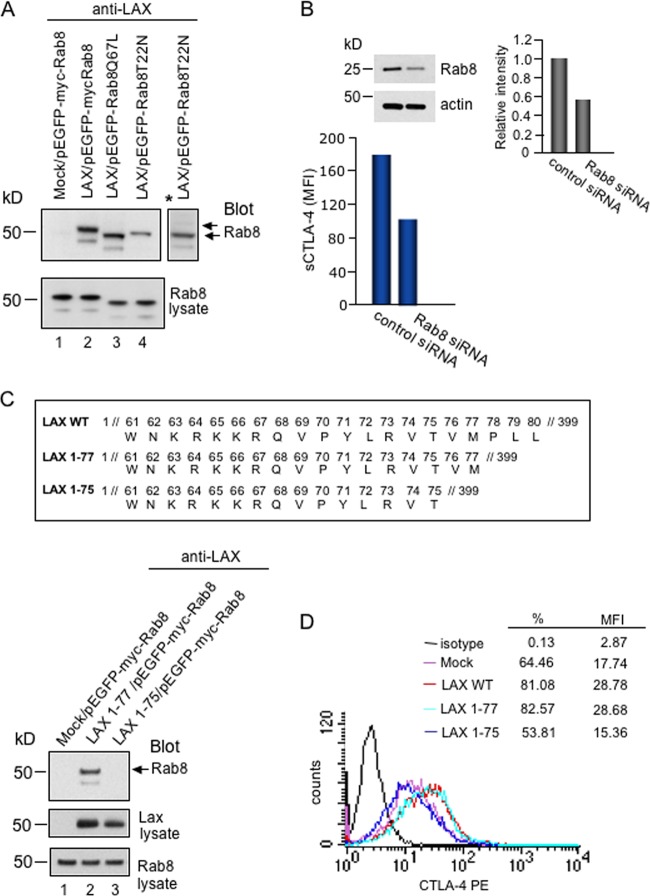
(A) Binding of LAX to wild-type and constitutively active Rab8. 293T cells cotransfected as indicated were immunoprecipitated with anti-LAX and blotted for Rab8 (upper panel). Anti-Rab8 blotting of cell lysates served as a loading control. The faster migration of Q67LRab8 and TN22Rab8 compared to wild-type Rab8 is due to the absence of the myc tag. *, overexposed version of lane 4. (B) Rab8 siRNA reduces CTLA-4 surface expression. Murine T cells were transfected with control or Rab8 siRNA and stimulated with ConA (2.5 μg/ml). After 3 days, cells were washed and stained for CTLA-4 with anti-CTLA-4–PE. A histogram shows the MFI of CTLA-4-positive cells. The inset shows reduced Rab8 expression in cells transfected with Rab8 siRNA, as demonstrated by blotting with anti-Rab8. (C) Rab8 binds to the N terminus of LAX. The upper panel shows a depiction of N-terminal LAX constructs. For the lower panel, 293T cells cotransfected as indicated were immunoprecipitated with anti-LAX and blotted for Rab8 (top panel). Cell lysates were blotted for LAX (middle panel) and Rab8 (bottom panel). (D) Reduced CTLA-4 surface expression in LAX1-75-transfected cells. Mock-, wild-type LAX-, LAX 1-77-, and LAX1-75-transfected DC27.10–CTLA-4 cells were stained with CTLA-4–PE and analyzed by fluorescence-activated cell sorting (FACS).

Next, it was important to assess whether LAX bound preferentially to the active GTP-bound form of Rab8 (Fig. 2A). LAX was therefore coexpressed in 293T cells with active (Q67L) and inactive (T22N) Rab8, followed by precipitation with anti-LAX and immunoblotting for Rab8. Strikingly, LAX associated with substantially more Q67LRab8 compared to inactive TN22Rab8 (Fig. 2A, upper panel, lane 3 versus lane 4). The faster migration of Q67LRab8 and TN22Rab8 compared to wild-type Rab8 is due to the absence of the myc-tag. An overexposed version of lane 4 (*) also revealed a lower fainter band present in the pulldowns of wild-type Rab8 and Q67LRab8, most likely representing a degradation product. Cell lysates blotted with anti-Rab8 revealed equal sample loading (Fig. 2A, lower panel, lanes 1 to 4). These data indicated that the active form of Rab8 binds with higher affinity to LAX than the inactive form.

Given this finding, we assessed whether Rab8 was needed for optimal CTLA-4 surface expression (sCTLA-4) (Fig. 2B). Primary T cells were transfected with Rab8 siRNA, followed by anti-CD3/CD28 coligation and analysis of sCTLA-4 72 h after transfection. Immunoblotting of cell lysates showed reduced Rab8 expression in Rab8 siRNA-transfected cells (Fig. 2B, upper panel). Concurrent with this was a substantial reduction in the mean fluorescence intensity (MFI) of sCTLA-4 compared to cells transfected with control siRNA (107 versus 182) as detected by flow cytometry (Fig. 2B, lower panel). These data indicated that CTLA-4 required Rab8 for optimal transport to the cell surface.

In an attempt to map the site of binding, mutants of LAX consisting of the N-terminal amino acids 1 to 75 or 1 to 77 were generated (Fig. 2C) and assessed for binding to Rab8. For this, 293T cells cotransfected with pEGFP-myc-Rab8 and mock, myc-LAX1-77, or myc-LAX1-75 were precipitated with anti-LAX and blotted for Rab8 (Fig. 2C, lower panel, lanes 1 to 3). LAX1-77 readily precipitated Rab8 (Fig. 2C, lower panel, lane 2), while LAX1-75 failed to associate with Rab8 (lane 3). Cell lysates blotted with anti-Rab8 showed equal sample loading (Fig. 2C, lower panel, lanes 1 to 3). Lax mutant 1-75 was slightly less expressed compared to mutant 1-77 (Fig. 2C, middle panel, lane 3 versus lane 2). Further, overexpression of LAX1-75 into DC27.10 cells stably transfected with CTLA-4 (DC27.10–CTLA-4) (20, 22) failed to support the increase in CTLA-4 surface expression observed with LAX WT (Fig. 2D). Although LAX WT increased the expression of CTLA-4 from 64.46 to 81.08%, LAX 1-75 showed a decrease from 64.46 to 53.8%. Together, these data indicated that the N-terminal region bound to Rab8, and this interaction played a role in the transport of CTLA-4 to the cell surface.

### Binding of LAX to TRIM in T cells.

**FIG 3:**
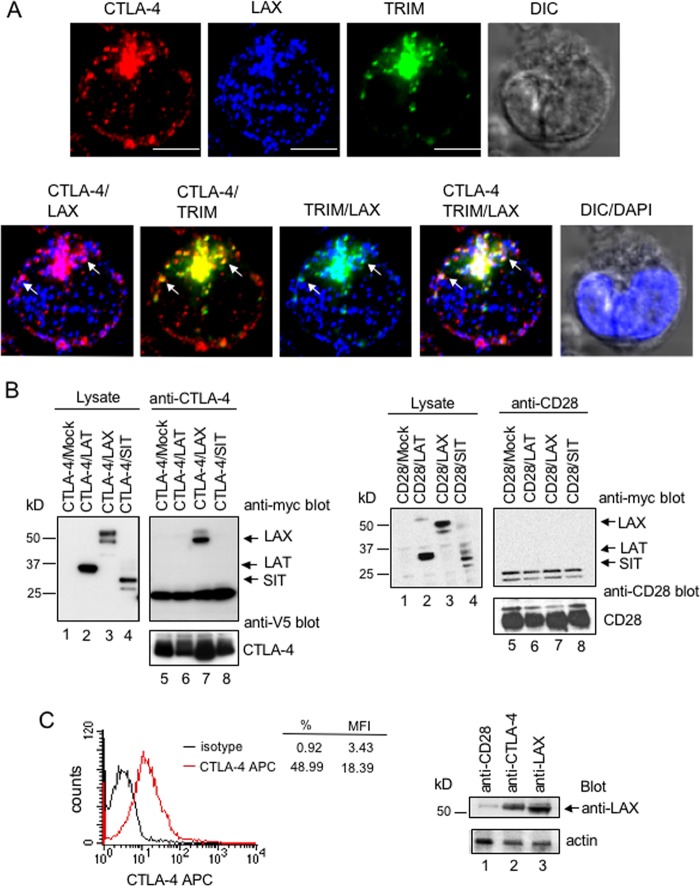
(A, top) Colocalization of CTLA-4, LAX, and TRIM in primary human T cells. Preactivated human T cells were stained with biotinylated anti-CTLA-4 plus streptavidin-Alexa Fluor 568 (CTLA-4 panel), anti-LAX plus anti-rabbit antibody–Alexa Fluor 488 (LAX panel), and anti-TRIM plus anti-mouse antibody–Alexa Fluor 647 (TRIM panel) and analyzed by confocal microscopy. (Bottom) Colocalization of CTLA-4/LAX (magenta), CTLA-4/TRIM (yellow), TRIM/LAX (cyan), and CTLA-4/TRIM/LAX (white) occurred mainly in the TGN area. Bars, 2.5 μm. (B) CTLA-4 binds to LAX but not LAT or SIT. In the left panel, 293T cells V5-CLTA-4 cotransfected as indicated were immunoprecipitated with anti-CTLA-4. Immunoblots were probed with anti-myc and anti-V5. For the detection of LAX, HRP–mouse anti-rabbit light chain, which did not react with the heavy chain of the precipitating CTLA-4 antibody, was used. In the right panel, 293T cells cotransfected as indicated were immunoprecipitated with anti-CD28. Immunoblots were probed with anti-myc and anti-CD28. For the detection of LAX, HRP–mouse anti-rabbit light chain, which did not react with the heavy chain of the precipitating CD28 antibody, was used. (C) For the left panel, activated murine T cells were stained with anti-CTLA-4-APC and analyzed by FACS for CTLA-4. For the right panel, activated murine T cells were lysed, immunoprecipitated with anti-CD28, anti-CTLA-4, and anti-LAX, and immunoblotted with anti-LAX. For the lower panel, cell lysates blotted with antiactin served as a loading control.

The binding of active Rab8 to LAX raised the question regarding the nature of the effector complex in T cells. We therefore further investigated the interaction of LAX with TRIM and CTLA-4 by initially using three-color immunofluorescence (Fig. 3A). Preactivated primary T cells were stained with biotinylated anti-CTLA-4 plus streptavidin-Alexa 568 (upper left panel), anti-LAX plus anti-rabbit antibody–Alexa 488 (upper middle panel) or anti-TRIM plus anti-mouse antibody–Alexa 647 (upper right panel) and analyzed by confocal microscopy. CTLA-4, LAX, and TRIM colocalized in the TGN and surrounding vesicles as viewed within a single confocal slice (lower panel). The only difference was the occasional staining of LAX in vesicles in other regions of the cells.

**FIG 4:**
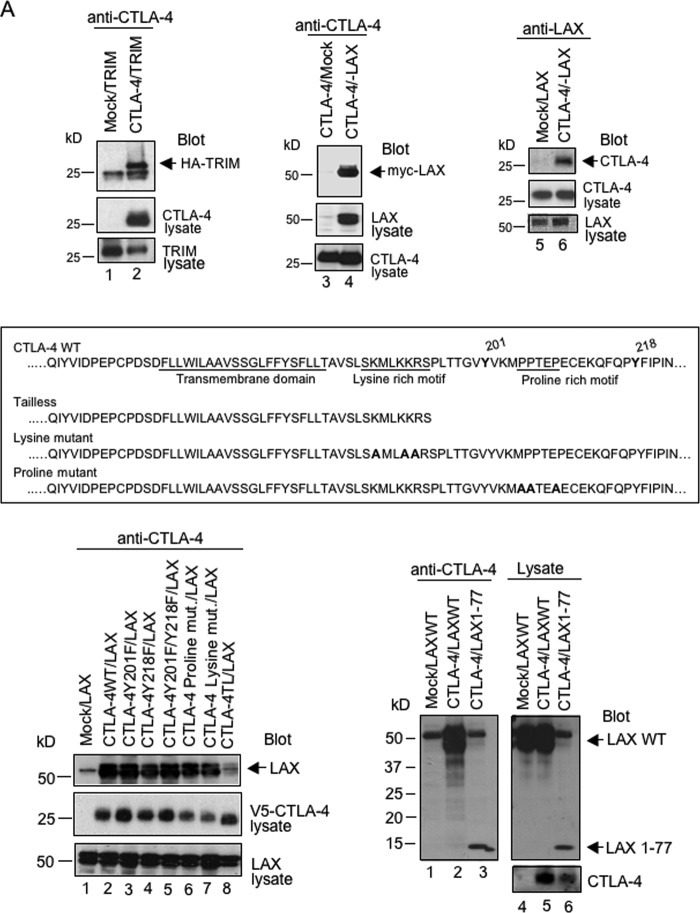

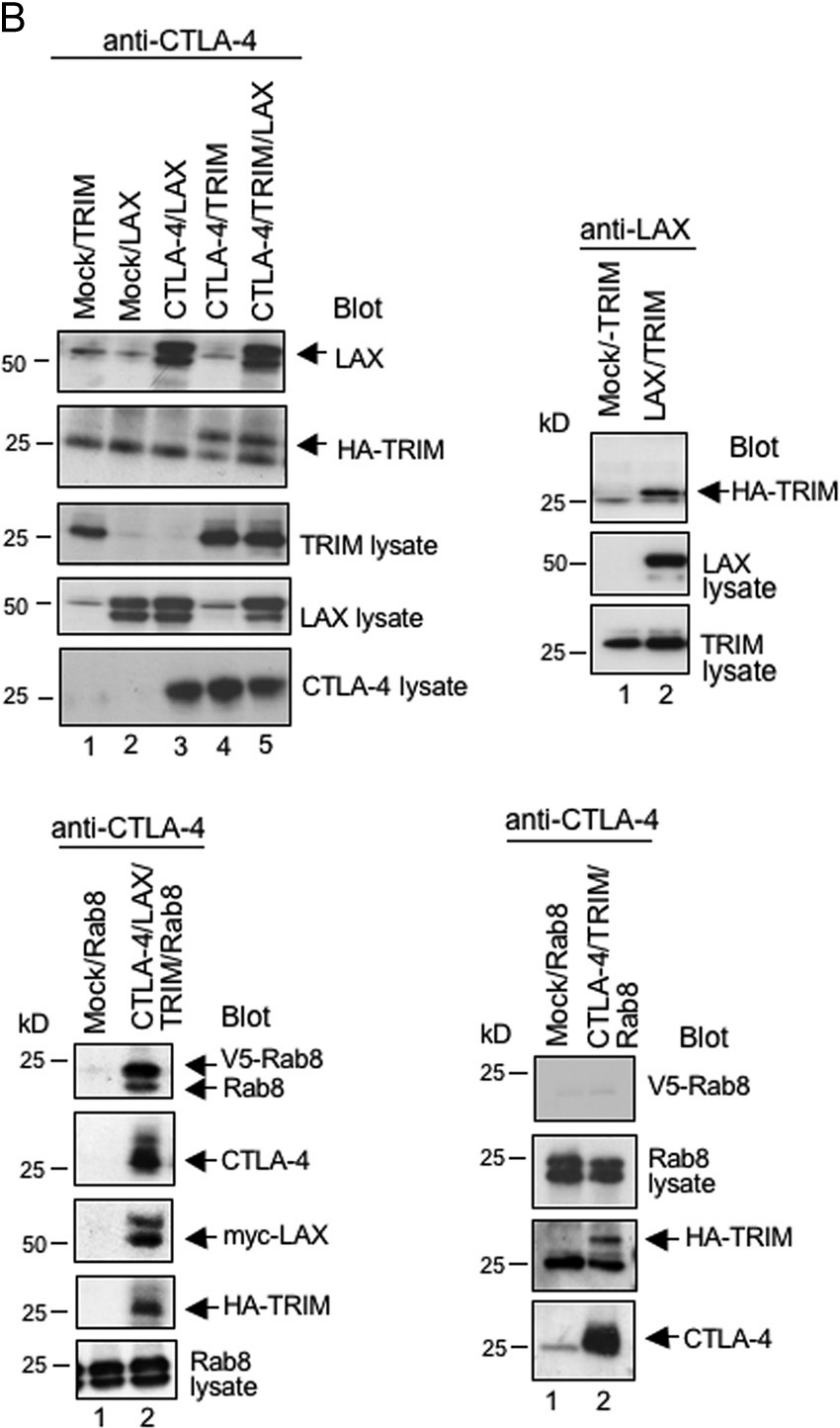
(A) Binding of LAX to CTLA-4 requires its cytoplasmic tail but is independent of its PI3K and AP-2 interaction. For the upper left panel, 293T cells that were mock and HA-TRIM cotransfected (lane 1) or CTLA-4 and HA-TRIM cotransfected (lane 2) were immunoprecipitated with anti-CTLA-4 and blotted with anti-HA. Anti-CTLA-4 (middle panel) or anti-HA (bottom panel) blotting of cell lysates served as a loading control. For the upper middle panel, 293T cells that were mock and CTLA-4 cotransfected (lane 3) or CTLA-4 and myc-LAX cotransfected (lane 4) were immunoprecipitated with anti-CTLA-4 and blotted with anti-myc. Anti-myc (middle panel) or anti-CTLA-4 (bottom panel) blotting of cell lysates served as a loading control. For the upper right panel, 293T cells that were mock and myc-LAX cotransfected (lane 5) or CTLA-4 and myc-LAX cotransfected (lane 6) were immunoprecipitated with anti-LAX and blotted with anti-CTLA-4. Anti-CTLA-4 (middle panel) or anti-myc (bottom panel) blotting of cell lysates served as a loading control. The middle panel shows wild-type CTLA-4, tailless CTLA-4, and point mutations of the tyrosine-, lysine-, and proline-rich motifs. For the lower left panel, 293T cells cotransfected as indicated (mut., mutant; TL, tailless) were immunoprecipitated with anti-CTLA-4 and blotted with anti-LAX (top panel). Blotting of cell lysates with V5 or LAX antibodies show equal expression of the various CTLA-4 mutants (middle panel) and wild-type LAX (bottom panel). The lower right panel shows the binding of LAX1-77 to CTLA-4. 293T cells cotransfected as indicated were precipitated with anti-CTLA-4 and blotted with anti-LAX (lanes 1 to 3). Cell lysates blotted with anti-LAX (upper panel) or anti-CTLA-4 (lower panel) served as a loading control (lanes 4 to 6). (B) The upper left panel shows that the overexpression of TRIM does not interfere in the binding of LAX to CTLA-4. Mock/TRIM (lane 1), Mock/LAX (lane 2), CTLA-4/LAX (lane 3), CTLA-4/TRIM (lane 4), or CTLA-4/TRIM/LAX (lane 5) were coexpressed in 293T cells, precipitated with anti-CTLA-4, and blotted for LAX (top panel) and TRIM (bottom panel). The third, fourth, and bottom panels show equal expression of TRIM, LAX, and CTLA-4 as revealed by blotting of cell lysates with anti-HA, anti-LAX, and anti-CTLA-4 antibodies, respectively. The upper right panel shows the binding of LAX to TRIM. 293T cells that were mock and HA-TRIM transfected (lane1) or myc-LAX and HA-TRIM transfected (lane 2) were immunoprecipitated with anti-LAX and blotted with anti-HA. Anti-myc (middle panel) or anti-HA (bottom panel) blotting of cell lysates served as a loading control. For the lower left panel, 293T cells that were V5-Rab8 and mock cotransfected (lane 1) or CTLA-4, HA-TRIM, and myc-LAX cotransfected (lane 2) were immunoprecipitated with anti-CTLA-4 and blotted with anti-Rab8 (top panel), anti-CTLA-4 (second panel), anti-LAX (third panel), and anti-HA (fourth panel). Anti-Rab8 blotting of cell lysates served as a loading control (bottom panel). For the detection of LAX, HRP–mouse anti-rabbit light chain, which did not react with the heavy chain of the precipitating CTLA-4 antibody, was used. For the detection of Rab8 and TRIM, HRP–goat anti-mouse Fc fragment specific IgG, which did not react with the light chain of the precipitating CTLA-4 antibody, was used. For the lower right panel, 293T cells V5-Rab8 and mock cotransfected (lane 1) or CTLA-4 and HA-TRIM cotransfected (lane 2) were immunoprecipitated with anti-CTLA-4 and blotted with anti-Rab8 (top panel), anti-HA (third panel), and anti-CTLA-4 (bottom panel). Anti-Rab8 blotting of cell lysates served as a loading control (second panel). For the detection of Rab8 and TRIM, HRP–goat anti-mouse Fc fragment-specific IgG, which did not react with the light chain of the precipitating CTLA-4 antibody, was used.

To test whether LAX could bind to CTLA-4, V5-tagged CTLA-4 was coexpressed with myc-tagged LAT, LAX, or SIT in 293T cells, followed by precipitation with anti-CTLA-4 and blotting with anti-myc antibody (Fig. 3B). Anti-CTLA-4 coprecipitated LAX (Fig. 3B, left panel, lane 7) but neither LAT (lane 6) nor SIT (lane 8). The adaptors were expressed at similar levels as detected by blotting of cell lysates (Fig. 3B, lanes 2 to 4). Further, unlike CTLA-4, CD28 failed to associate with LAX (Fig. 3B, right panel, lane 7). In addition to cells transfected with LAX, CTLA-4 also associated with endogenous LAX in preactivated primary T cells (49% of these activated T cells expressed CTLA-4) (Fig. 3C, left histogram) as detected by anti-LAX blotting (right panel, lane 2). Cell lysates blotted with antiactin served as a loading control (lower panel). Further, as an additional control, a comparison of cells transfected with CTLA-4 and HA-TRIM or myc-LAX showed that anti-CTLA-4 precipitated both TRIM (Fig. 4A, upper left panel, lane 2) and LAX (upper middle panel, lane 4). The interaction between LAX and CTLA-4 could also be demonstrated by immunoprecipitating LAX and blotting for CTLA-4 (Fig. 4A, upper right panel, lane 6). It was next of interest to assess whether binding of LAX to CTLA-4 requires its cytoplasmic tail or its interaction with PI3K or the clathrin adaptor AP-2. Several point mutations of CTLA-4 and tailless (TL) CTLA-4 (Fig. 4A, middle panel) were coexpressed with LAX in 293T cells, followed by precipitation with anti-CTLA-4 and blotting with anti-LAX antibody (lower left panel). Neither mutations of the two tyrosine-based motifs of CTLA-4 nor mutations of its proline- and lysine-rich motifs prevented the interaction between LAX and CTLA-4 (Fig. 4A, middle panel, lanes 3 to 7 versus lane 2). Although the cytoplasmic domain of CTLA-4 is needed for binding to LAX (lane 8), it is independent of its interaction with PI3K (Fig. 4A, middle panel, lanes 3 and 5) and AP-2 (lane 3). Middle and lower panels show equal expression of the various CTLA-4 mutants and wild-type LAX, respectively. To assess whether the Rab8 binding LAX1-77 was sufficient to associate with CTLA-4, wild-type LAX and LAX1-77 were coexpressed with CTlA-4 in 293T cells, immunoprecipitated with anti-CTLA-4 and blotted with anti-LAX antibody. Like wild-type LAX, mutant LAX1-77 could precipitate CTLA-4 (Fig. 4A, lower right panel, lane 3 versus lane 2).

It was next of interest to assess whether overexpression of TRIM would interfere in the binding of LAX to CTLA-4. To test this, CTLA-4, TRIM and LAX were coexpressed in 293T cells, precipitated with anti-CTLA-4 and blotted for LAX and TRIM (Fig. 4B, upper left panel). The amount of CTLA-4-associated LAX was comparable to that precipitated with anti-CTLA-4 from cells transfected with CTLA-4 and LAX in the absence of TRIM (Fig. 4B, upper left panel, lane 5 versus lane 3); the middle, upper, and lower panels in this portion of Fig. 4B show equal expression of TRIM, LAX, and CTLA-4, respectively. This finding indicates that LAX and TRIM in the complex are complementary and not competitive.

Notably, anti-LAX also coprecipitated TRIM from cells transfected with HA-TRIM and myc-LAX (Fig. 4B, upper right panel, lane 2 versus lane 1). These data indicated that in addition to the association of LAX with Rab8, LAX also bound to CTLA-4 and TRIM. To prove this further, CTLA-4 was cotransfected with LAX, TRIM, and Rab8 into 293T cells, precipitated with anti-CTLA-4, and blotted for Rab8 (Fig. 4B, lower left panel, lane 2). Under this condition, anti-CTLA-4 could precipitate Rab8, suggesting that the LAX/Rab8 complex is important for CTLA-4 transport to the surface of T cells. As a control, cotransfection of CTLA-4, TRIM, and Rab8, followed by immunoprecipitation with anti-CTLA-4 and blotting for Rab8, failed to show the interaction between CTLA-4 and Rab8 (Fig. 4B, lower right panel, lane 2) but did show binding to TRIM (upper lower panel, lane 2).

### LAX modulates CTLA-4 surface expression and IL-2 production.

**FIG 5:**
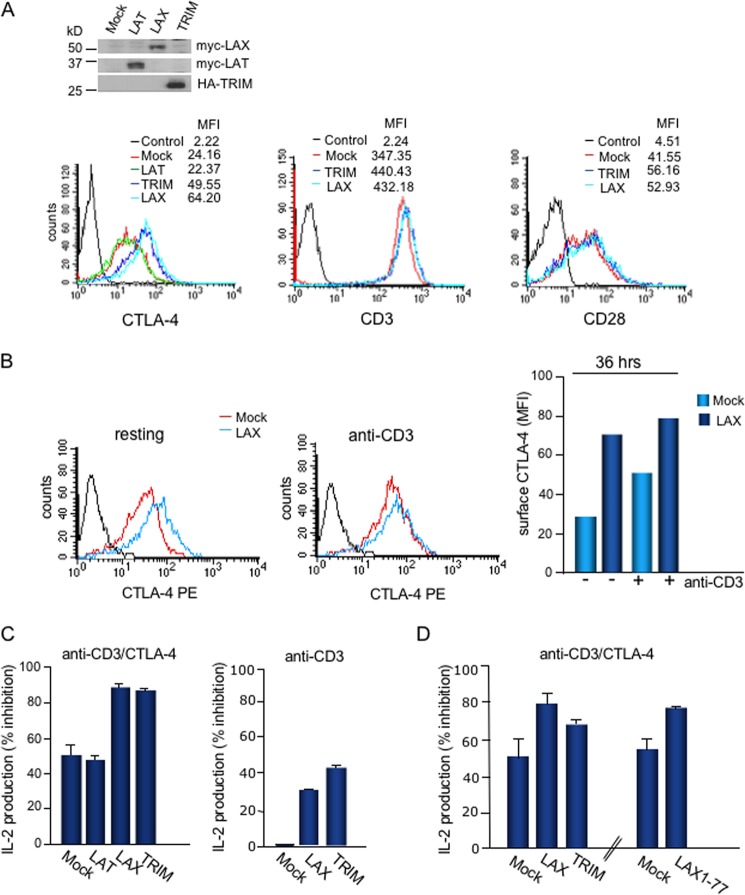
LAX upregulates CTLA-4 surface expression. (A) Mock-, LAT-, TRIM-, or LAX-transfected DC27.10–CTLA-4 cells were stained with CTLA-4–PE, CD3-PE, and CD28-PE, and the surface expression of CTLA-4, CD3, and CD28 was analyzed by FACS. The inset shows the expression levels of transfected LAX, LAT, and TRIM. (B) Mock- or LAX-transfected DC27.10–CTLA-4 cells were either left untreated (left panel) or stimulated with anti-CD3 (1 μg/ml, middle panel). At 36 h after stimulation, cells were stained with anti-CTLA-4–PE, and the surface expression of CTLA-4 was analyzed by FACS. In the right panel, a histogram shows the MFIs for surface CTLA-4 in resting and stimulated mock- and LAX-transfected cells. (C) LAX upregulates CTLA-4 function. In the left panel, Mock-, LAT-, LAX-, or TRIM-transfected DC27.10–CTLA-4 cells were stimulated with plate-bound anti-CD3 (1 μg/ml) or anti-CD3/CTLA-4 (1 μg/ml/10 μg/ml.) In the right panel, mock-, LAX-, or TRIM-transfected DC27.10–CTLA-4 cells were stimulated with plate-bound anti-CD3 (1 μg/ml). After 24 h, the supernatant was taken and analyzed for IL-2 by ELISA. The bar graph shows means ± the standard deviations (SD; *n* = 2). (D) Primary murine T cells transfected with mock, LAXWT, LAX1-77, or TRIM were stimulated with plate-bound anti-CD3 (2.5 μg/ml) or anti-CD3/CTLA-4 (2.5 and 10 μg/ml, respectively) and, 48 h later, intracellular staining for IL-2 was performed. The bar graph shows the means ± the SD (*n* = 2).

Given the connection between Rab8 and LAX, as well as between CTLA-4 and LAX, it was of interest to assess whether LAX could modulate CTLA-4 surface expression. DC27.10–CTLA-4 cells that were mock, LAT, TRIM, or LAX transfected were initially stained and analyzed for surface CTLA-4 using anti-CTLA-4–PE in the absence of anti-CD3 ligation (Fig. 5A). LAX expression had a marked effect by increasing the MFI of sCTLA-4 from 24 to 64; TRIM increased the MFI for sCTLA-4 from 24 to 50. Importantly, as a negative control, expression of LAT had no effect on the MFI or the percent expression of sCTLA-4. Further, transfection of TRIM or LAX did not alter CD3 or CD28 surface expression (Fig. 5A, middle and right panels). The levels of transfected LAX, LAT, and TRIM are shown, as determined by immunoblotting (upper inset). These observations indicated that LAX had the ability to selectively increase CTLA-4 surface expression in the absence of anti-CD3 ligation. LAX modulation of sCTLA-4 occurred independently of its reported effect on TCR signaling (36).

Further, LAX cooperated with anti-CD3 ligation to increase sCTLA-4 (Fig. 5B). The ability of anti-CD3 stimulation to induce CTLA-4 expression has been well documented (23). Mock- or LAX-transfected DC27.10–CTLA-4 cells were either left untreated (Fig. 5B, left panel) or stimulated with plate-bound anti-CD3 (1 μg/ml) (Fig. 5B, middle panel). After 36 h, the cells were stained and analyzed for sCTLA-4 expression. LAX increased the MFI for sCTLA-4 from 28 to 71, whereas anti-CD3 alone increased the MFI from 28 to 45 (right histogram). The combination of anti-CD3 and LAX cooperated to increase the MFI for sCTLA-4 to 77. These data indicated that LAX and anti-CD3 effectively cooperate to induce high levels of surface CTLA-4 on T cells.

Importantly, this increase in expression of surface CTLA-4 induced by LAX resulted in a profound increase on the level of inhibition of IL-2 production when expressed with coligation by anti-CD3 and anti-CTLA-4 (Fig. 5C, left panel). Although anti-CTLA-4 inhibited IL-2 production by 45 to 50% in mock- or LAT-transfected cells, cells expressing LAX or TRIM showed inhibition of IL-2 production by 80 to 90%. In contrast, as a control, LAX and TRIM expression inhibited anti-CD3-induced IL-2 production by 32 and 43%, respectively (middle panel). This is in accordance with a previous report demonstrating that LAX can inhibit TCR signaling (36), although the effect with anti-CD3 alone was considerably lower compared to the coligation of CTLA-4 (i.e., 32% versus 89%). The increased inhibitory effect on IL-2 production mediated by anti-CD3/CTLA-4 coligation could also be demonstrated in primary T cells transfected with LAX and TRIM (Fig. 5D). Notably, cells transfected with LAX1-77 led to an inhibition in IL-2 production comparable to that mediated by LAX WT and TRIM.

Our data therefore show that while LAX can exert a partial inhibitory effect on TCR signaling, it cannot account for the more robust inhibition seen with the increased level of CTLA-4 expression and inhibition on T cells. These findings demonstrate that LAX can exert an inhibitory effect on T-cell activation by regulating the expression of CTLA-4 on the surface of T cells.

**FIG 6:**
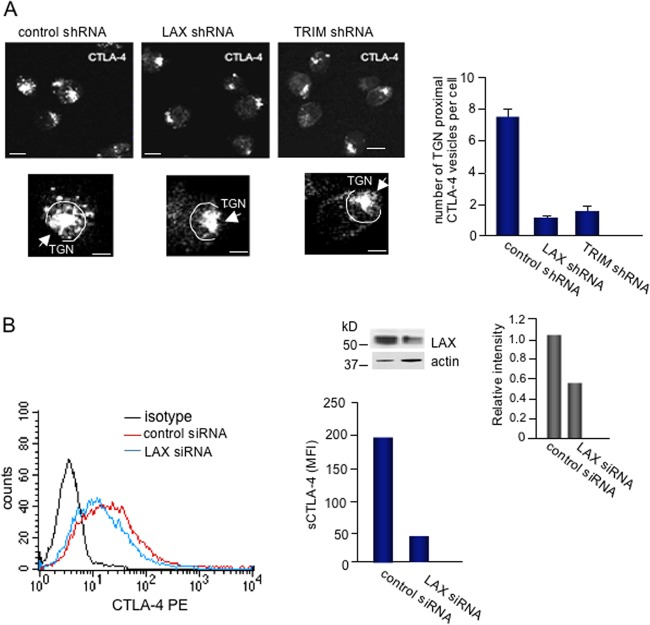
Reduction of TGN-proximal CTLA-4-containing vesicles in cells transfected with shRNAs. (A) For the upper panel, DC27.10–CTLA-4 cells were transfected with control shRNA, LAX shRNA, and TRIM shRNA and stained with anti-CTLA-4–Texas Red 3 days after transfection (left panel). The presence of CTLA-4-containing vesicles were analyzed by confocal microscopy and ImageJ. Bars, 10 μm; bars in the enlarged images, 5 μm. The circled area in the enlarged images indicates the area (2.5 μm) in which TGN-proximal vesicles were counted. In the right panel, a histogram shows the numbers of CTLA-4 vesicles from cells transfected with control, LAX, and TRIM shRNA (*n* > 30 cells for each condition). (B) LAX siRNA reduces CTLA-4 surface expression. Murine T cells were transfected with control or LAX siRNA and stimulated with concanavalin A (2.5 μg/ml). After 3 days, the cells were washed, stained for CTLA-4 with anti-CTLA-4–PE, and analyzed by FACS. A histogram shows the MFIs of CTLA-4-positive cells. For the inset, reduced LAX expression in cells transfected with LAX siRNA is demonstrated by blotting with anti-LAX.

Conversely, a reduction in LAX or TRIM expression by shRNA reduced the presence of CTLA-4 vesicles and cell surface expression of the coreceptor (Fig. 6). DC27.10–CTLA-4 cells were transfected with LAX shRNA, stained for intracellular CTLA-4, and analyzed by confocal microscopy (Fig. 6A). A vesicle within 2.5 μm of the TGN was defined as TGN-proximal vesicle. Clearly, LAX shRNA reduced the number of CTLA-4-containing vesicles per cell, with the majority being localized in the TGN. Further, transfection of primary T cells with LAX siRNA showed a 4-fold reduction of the MFI for CTLA-4 surface expression (Fig. 6B). Reduced expression of LAX in LAX siRNA-transfected cells was detected by blotting of cell lysates (Fig. 6A, upper inset). Overall, these data indicated that TRIM and LAX regulate the formation of TGN-proximal CTLA-4-containing vesicles needed for optimal CTLA-4 surface expression and increased inhibition of T-cell responses.

### LAX modulates CTLA-4 and MTOC polarization.

**FIG 7:**
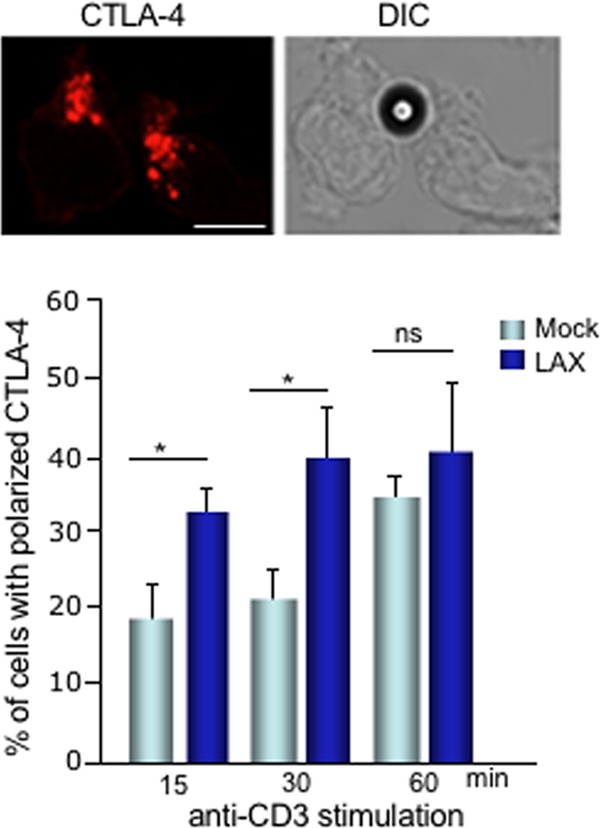
LAX increases the polarized release of CTLA-4. DC27.10–CTLA-4 cells were mock or LAX transfected and stimulated with anti-CD3-coated beads for the indicated periods of time. The cells were then fixed, permeabilized, stained with anti-CTLA-4–Alexa Fluor 568, and analyzed by confocal microscopy. Cells with polarized CTLA-4 vesicles in a cell-bead contact area of 4 μm were counted. The upper panel shows an image with polarized CTLA-4 toward the contact region between T cells and the anti-CD3-coated beads. Bar, 10 μm. The histogram below shows the percentages of cells with polarized CTLA-4 (*n* = 3; 120 to 150 cells/experiment). The means ± the SD are displayed. Differences between means were tested by using two-tailed unpaired Student *t* test (*, *P* < 0.05 [considered significant]; ns, not significant).

Previous studies have shown that anti-CD3 ligation induces the polarization of CTLA-4 to the contact region of T cells (23, 56). To assess whether LAX could also affect the polarized release of CTLA-4 in response to anti-CD3 ligation, mock- or LAX-transfected DC27.10–CTLA-4 cells were stimulated for various periods of time with anti-CD3- or isotype-coated Dynabeads. Cells were then fixed, permeabilized, stained with anti-CTLA-4, and analyzed by confocal microscopy (Fig. 7A). CTLA-4 polarized vesicles were defined by being within 4 μm from the cell-bead contact area. Compared to mock-transfected cells, LAX expression accelerated and increased the polarization of CTLA-4 to the contact region between T cells and anti-CD3-coated beads (lower histogram). This was evident as early as 15 to 30 min. By 60 min, the polarization of CTLA-4 in mock-transfected cells induced by anti-CD3 alone occurred at levels similar to those seen with the LAX-transfected cells.

**FIG 8:**
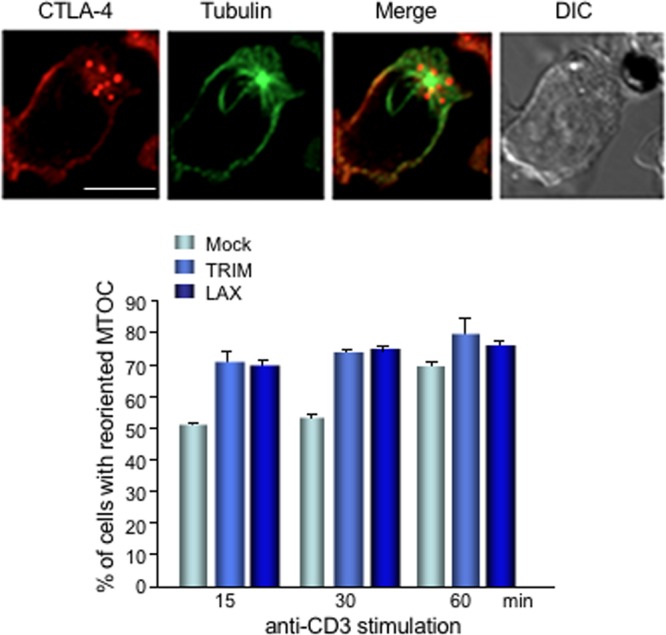
TRIM and LAX accelerate the MTOC reorientation. DC27.10–CTLA-4 cells were mock, TRIM, or LAX transfected and stimulated with anti-CD3-coated beads for the indicated periods of time. The cells were then fixed, permeabilized, and stained with anti-CTLA-4–Alexa 568 and anti-α-tubulin–Alexa Fluor 488. The cells were analyzed by confocal microscopy (CTLA-4, red; MTOC [microtubules], green). Bar, 10 μm. A cell was considered positive for MTOC reorientation if its MTOC was juxtaposed to a bead in contact with the cell (upper panel). Cells with polarized MTOC toward the cell-bead contact area of 3 μm were counted. The histogram shows the percentages of cells with reoriented MTOC stimulated for the indicated time points (*n* = 2; 50 to 70 cells/sample).

The polarized release of CTLA-4 to the anti-CD3 interface in turn has previously been correlated with a repositioning of the MTOC in T cells (56). The increase in polarized CTLA-4 could therefore be related to an increase in the polarization of the MTOC. DC27.10–CTLA-4 cells were therefore mock, LAX, or TRIM transfected, stimulated with anti-CD3, and then fixed, permeabilized, and stained with anti-alpha-tubulin. In activated cells, the MTOC was clearly oriented toward attached anti-CD3-coated beads, as revealed by confocal microscopy (Fig. 8, upper panel). Polarized MTOC was defined by being within 3 μm from the cell-bead contact area. Notably, 70% of cells transfected with LAX or TRIM had the MTOC reoriented to the interface with beads at 15 min anti-CD3 stimulation compared to only 50% of mock-transfected cells. This difference could also be observed at 30 min stimulation. Only after 60 min of stimulation mock-transfected cells reached the percentage of cells with reoriented MTOC observed in LAX- or TRIM-transfected cells (Fig. 8, lower histogram). Together, our data demonstrated that LAX and TRIM regulate the MTOC reorientation and the polarization of CTLA-4 to the contact site of TCR engagement for increased CTLA-4 surface expression. This is in accordance with the well-established role of Rab8 in modulating polarized membrane transport to cell surfaces (48, 49).

## DISCUSSION

Although CTLA-4 is located mostly in the intracellular *trans*-Golgi network, the mechanism by which it is transported to the cell surface had been unclear. In the present study, we have identified a novel complex comprised of TRIM and related LAX that in turn binds to GTP bound Rab8 for post-Golgi transport to the cell surface. LAX bound via its N terminus to active GTP-Rab8, as well as the cytoplasmic tail of CTLA-4. TRIM required LAX for binding to Rab8 in a complex. Wild-type LAX or its N terminus (residues 1 to 77) increased CTLA-4 surface expression, whereas siRNAs of Rab8 or LAX or disruption of LAX/Rab8 binding reduced the numbers of CTLA-4-containing vesicles and its coreceptor surface expression. Our findings identify a novel effector of Rab8 for the transport of CTLA-4 from the *trans*-Golgi network to the surfaces of T cells for the mediation of immune suppression.

Rab8 has previously been shown to mediate the trafficking of newly synthesized proteins from the TGN to the plasma membrane (55). GTP-bound Rab8 has been reported to bind to optineurin (57), MAP4K2 (52), FIP-2 (53), and rab8ip/GC kinase (52). However, despite high levels of expression in T cells, no binding partners or effectors of Rab8 have been described. We have identified here a GTP-Rab8-LAX complex that implicates LAX as an effector of Rab8 in the regulation of CTLA-4 surface expression. LAX bound to GTP-Rab8 via its N terminus and the disruption of LAX/Rab8 binding profoundly reduced the formation of CTLA-4-containing vesicles and the expression of CTLA-4 on the surfaces of T cells. LAX was bound to CTLA-4, acting as a bridge between Rab8 and the coreceptor for its transport. As a control, LAX associated with CTLA-4 but not with the related CD28. Conversely, CTLA-4 bound to LAX but not to the other type 1 protein, LAT, that mediates TCR signaling. The shRNA knockdown of LAX markedly reduced the numbers of CTLA-4-containing vesicles proximal to the TGN and CTLA-4 surface expression. In contrast, LAX overexpression increased CTLA-4 surface expression, without affecting the expression of the TCR or CD28. The LAX-induced increase in CTLA-4 expression led to an inhibition of T-cell activation upon coligation of CTLA-4 with the TCR. This finding is in accordance with a reported role of the adaptor in the inhibition of T-cell activation (36). Overall, LAX is a novel effector of the Rab8 pathway that controls the transport and surface expression of CTLA-4 for the inhibition of T-cell responses.

Previous studies have underscored the importance of Rab8 to the organization of the endocytic compartment (44) and the remodeling of actin and microtubules for directed membrane transport to cell surfaces (48, 49). Despite its high expression in T cells and the fact that it can interact with effectors in other cell types, no immune cell-specific binding effectors of Rab8 have been identified. Our findings therefore identify for the first time LAX as an immune cell-specific adaptor protein that bound preferentially to the active GTP-bound form of Rab8 and, as such, is likely to serve as an effector of Rab8 in T cells. Our findings suggest that LAX functions as a central coordinator by bridging Rab8 with the other LAX-associated proteins, TRIM and CTLA-4. Increased LAX expression in turn increased CTLA-4-containing TGN-proximal vesicles, cell surface expression, and inhibition of TCR-induced IL-2 production. At the same time, siRNA knockdown of Rab8 or a LAX mutant defective in binding to Rab8 impaired the expression of CTLA-4 at the cell surface. Collectively, these findings point to a central role for Rab8 and is effector protein, LAX, in the regulation of the transport of CTLA-4 to the surfaces of T cells for inhibition of the immune response. These data are of potential importance to the field of antitumor immunology given the successful application of anti-CTLA-4 (Ipilimumab) in the treatment of late-stage melanoma (58).

**FIG 9:**
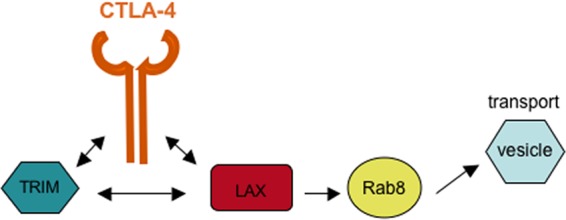
CTLA-4 forms a multimeric complex comprised of TRIM, LAX, and Rab8. TRIM and LAX bind to the cytoplasmic tail of CTLA-4, while LAX binds via its N terminus to active GTP-Rab8. TRIM requires LAX for binding to Rab8 in a complex. LAX functions as a central coordinator by bridging Rab8 with the other LAX-associated proteins, TRIM and CTLA-4. These findings identify a novel CTLA-4/TRIM/LAX/Rab8 effector complex in the transport of CTLA-4 to the surfaces of T cells.

We previously reported that CTLA-4 also interacts with TRIM, another type 1 transmembrane adaptor that is immune cell specific and whose downregulation impaired the expression of CTLA-4 (22). LAX therefore shares with TRIM a role in the early stage of TGN-derived CTLA-4-containing vesicle formation for transport to the cell surface. However, in contrast, LAX was able to bind to both TRIM and CTLA-4 in a specific manner. For example, it did not bind to LAT or CD28 in coexpression and coprecipitation assays. LAX differed from TRIM in that it had the binding properties of a central coordinator of the CTLA-4 transport process. It had the unusual ability to bind directly to Rab8 in addition to CTLA-4 and TRIM, as seen by coexpression in nonlymphoid cells. Rab8 is a key marker for vesicle trafficking from the TGN to the plasma membrane. This observation, together with the binding of LAX to TRIM and CTLA-4, suggests that it can operate as a coordinator in the formation of a complex for proper CTLA-4 transport to the plasma membrane (Fig. 9).

Increased CTLA-4 expression by LAX in turn led to increased inhibition of IL-2 production by anti-CD3/CTLA-4 coligation. In accordance with the reported role of LAX in the inhibition of T-cell activation (36), LAX expression reduced IL-2 production by ca. 30%, part of which may be related to increased CTLA-4 expression. However, at the same time, the combined effects of LAX and anti-CD3/CTLA-4 inhibited IL-2 production by more than 80%. This was in keeping with the ability of LAX to increase CTLA-4 expression on >85% of cells, which is unprecedented in the literature. Our data therefore show that while LAX can exert a partial effect on TCR signaling, it cannot account for the more robust inhibition seen with the increased level of CTLA-4 expression and inhibition on T cells. These findings show that LAX can exert a discrete inhibitory effect on T-cell activation by regulating the expression of CTLA-4 on the surfaces of cells. Further, T cells from LAX knockout mice were hyperstimulated by anti-CD3 via an unknown mechanism. Our data suggest that the loss of LAX may exert its effect by reducing CTLA-4 expression and thus predisposing T cells to increased activation due to the absence of the negative regulator (36).

In accordance with the well-established role of Rab8 in modulating polarized membrane transport through the reorganization of actin and microtubules to cell surfaces (48, 49), LAX expression was found to modulate the polarization of the MTOC and CTLA-4 to the contact regions of T cells with anti-CD3-coated beads. This therefore provides a potential molecular basis to explain the previous observation that CTLA-4 polarizes toward the sites of TCR engagement and immunological synapse of T cells (23, 56). During antigen presentation, the MTOC reorients itself from the trailing region of the cell to the area between the nucleus and the TCR contact region. The Rab8/LAX pathway is likely to mediate the directional release since overexpression of LAX enhanced the reorientation of the MTOC and the polarized release of CTLA-4 to the contact site on anti-CD3-coated beads. It is unclear in the literature whether the formation of vesicles can influence the signals that induce the reorientation of the MTOC for the polarized release of intracellular molecules. The ability of LAX to facilitate the reorientation of the MTOC would be compatible with this interpretation or the possibility that LAX independently affects the MTOC by other mechanisms. In either case, the combined effects of increased CTLA-4/LAX/Rab8 complex formation, and the enhanced polarization of the MTOC would act to facilitate the optimal release of CTLA-4 to the surfaces of cells.

Overall, our results identified a novel Rab8-LAX complex that mediates CTLA-4 transport to the surfaces of T cells. Future work will be needed to determine whether this pathway operates in conjunction with other signaling events. For example, CTLA-4, TRIM, and LAX all possess an YXXM-binding motif for PI3K. A report by Simonsen et al. highlights the importance of coregulation between Rab family GTPases and phosphoinositides (59). Activated Rab5 on endocytosed vesicles recruits phosphatidylinositol-3-phosphate [PtdIns(3)P], whereas Rab8 recruits PtdIns(4)P at the TGN and secretory vesicles by regulating the phosphoinositide phosphatase OCRL (oculocerebrorenal syndrome of Lowe) (60). Both Rab family GTPases and phosphoinositides are needed for the polarized transport of secretory vesicles (61, 62). Further, it was recently shown that p85 of PI3K binds and regulates the phosphatase PTEN (phosphatase and tensin homologue), providing a link from PI3K to OCRL (63).
